# High-performance PBPK model for predicting CYP3A4 induction-mediated drug interactions: a refined and validated approach

**DOI:** 10.3389/fphar.2025.1521068

**Published:** 2025-02-26

**Authors:** Cheng-Guang Yang, Tao Chen, Wen-Teng Si, An-Hai Wang, Hong-Can Ren, Li Wang

**Affiliations:** ^1^ Department of General Surgery, Tongren Hospital, Shanghai Jiao Tong University School of Medicine, Shanghai, China; ^2^ Shanghai PharmoGo Co., Ltd., Shanghai, China; ^3^ Department of Joint Surgery, Zhengzhou Orthopaedic Hospital, Zhengzhou, China; ^4^ Neurology Department, The First Affiliated Hospital of Zhengzhou University, Zhengzhou, China; ^5^ Department of Drug Discovery and Development, GenFleet Therapeutics (Shanghai) Inc., Shanghai, China

**Keywords:** drug interactions, rifampicin, CYP3A enzyme, PBPK, pharmacokinetics

## Abstract

**Introduction:**

The cytochrome P450 enzyme 3A4 (CYP3A4) mediates numerous drug-drug interactions (DDIs) by inducing the metabolism of co-administered drugs, which can result in reduced therapeutic efficacy or increased toxicity. This study developed and validated a Physiologically Based Pharmacokinetic (PBPK) model to predict CYP3A4 induction-mediated DDIs, focusing on the early stages of clinical drug development.

**Methods:**

The PBPK model for rifampicin, a potent CYP3A4 inducer, was developed and validated using human pharmacokinetic data. Subsequently, PBPK models for ‘victim’ drugs were constructed and validated. The PBPK-DDI model’s predictive performance was assessed by comparing predicted area under the curve (AUC) and maximum concentration (C_max_) ratioswith empirical data, using both the 0.5 to 2-fold criterion and theGuest criteria.

**Results:**

The rifampicin PBPK model accurately simulated human pharmacokinetic profiles. The PBPK-DDI model demonstrated high predictive accuracy for AUC ratios, with 89% of predictions within the 0.5 to 2-fold criterion and 79% meeting the Guest criteria. For Cmax ratios, an impressive 93% of predictions were within the acceptable range. The model significantly outperformed the static model, particularly in estimating DDI risks associated with CYP3A4 induction.

**Discussion:**

The PBPK-DDI model is a reliable tool for predicting CYP3A4 induction-mediated DDIs. Its high predictive accuracy, confirmed by adherence to evaluation standards, affirms its reliability for drug development and clinical pharmacology. Future refinements may further enhance its predictive value.

## Graphical Abstract

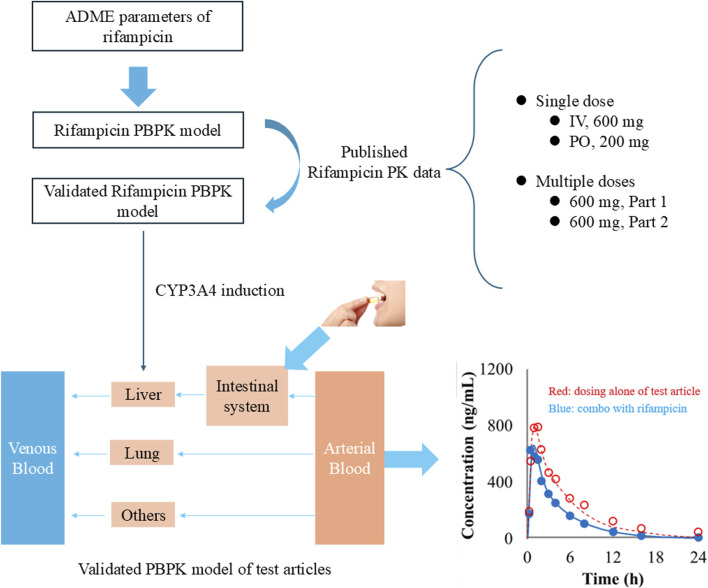

## Introduction

In the realm of pharmacology and drug development, interactions between concurrently administered drugs have emerged as a critical focus due to their profound impact on patient safety and therapeutic efficacy (Prueksaritanont et al., 2013). Among these, interactions mediated by the CYP3A4 are of particular concern, given its central role in metabolizing a broad spectrum of therapeutic agents. The CYP3A4-mediated potential to either induce or inhibit the metabolism of concurrently administered drugs can profoundly modify their pharmacokinetic profiles, which may consequently elevate the risk of adverse events due to drug-drug interactions. This sometimes can lead to the withdrawal of the medication from the market (Smith and Schmid, 2006).

The induction of CYP3A4 can accelerate the clearance of co-administered drugs, potentially leading to suboptimal therapeutic effects (Skolnick et al., 1976). Additionally, this induction may result in an increased exposure to toxic metabolites, thereby raising safety concerns due to the potential toxicity (Gramec Skledar et al., 2016; Murphy et al., 2005). Therefore, the accurate prediction of CYP3A4-mediated interactions is essential for ensuring the safe and effective use of new molecular entities in drug development.

The PBPK model is particularly relevant in scenarios such as dose optimization for individualized therapy, prediction of drug-drug interactions, and evaluation of the impact of disease states on drug disposition (Chang et al., 2023; Rowland Yeo et al., 2024). It is also instrumental in extrapolating preclinical data to human conditions and in guiding clinical trial design by simulating various dosing regimens and schedules (Seo et al., 2022; Statelovaet al., 2023). PBPK models have shown promise as predictive tools for DDIs (Yamashita et al., 2013). While there have been numerous individual case reports attesting to the predictive capabilities of PBPK models, particularly for CYP3A4 induction-related DDIs, a systematic assessment of these models across a broader spectrum of DDIs is lacking. Addressing this gap is essential for refining predictive methodologies and enhancing our understanding of the models’ implications for drug development and clinical practice.

Building on our previous work, which successfully developed and validated a PBPK-DDI model for CYP3A4 inhibition-mediated DDIs (Jiang et al., 2023; Ren et al., 2021), the current study addresses the need for robust predictive methodologies for CYP3A4 induction-mediated DDIs, especially during the early clinical stages of drug development. Our approach involves developing a PBPK-DDI model that integrates a comprehensive dataset of substrates for PBPK model development of substrates and their documented interaction outcomes with strong CYP3A4 inducer (rifampicin). This PBPK-DDI model prioritizes the use of empirical data from *in vitro* and clinical studies, employing mechanistic predictions only for parameters that are difficult to measure experimentally. By comparing the model’s predictions with documented DDIs, we aim to validate its efficacy in anticipating the clinical impact of CYP3A4-mediated interactions, thereby contributing to a more accurate and reliable prediction tool for drug developers and clinicians.

## Methods

**FIGURE 1:**
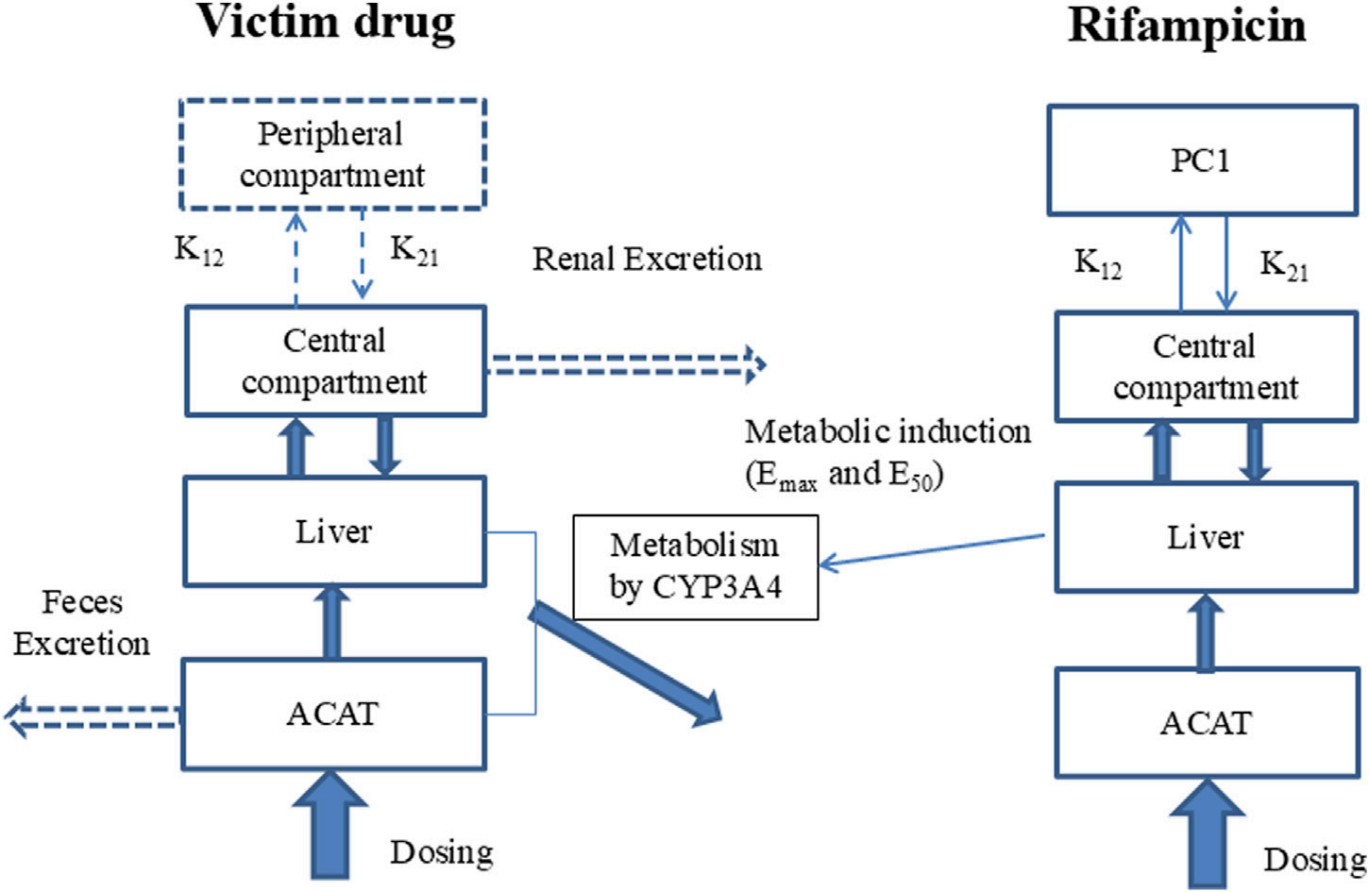
Model of PBPK-DDI. Note: Compartments and arrows with dash lines represent the options in the model. PC1, peripheral compartment 1; PC2, peripheral compartment 2; K_12_, transfer rate constant from central compartment to peripheral compartment; K_21_, transfer rate constant from peripheral compartment to peripheral central compartment; V_max_ and K_m_, the constants of Michaelis-Menten reaction kinetics mediated by CYP3A4, which was assumed to be the unique metabolism rout; EC_50_ and E_max_ are the induction potency and magnitude to CYP3A4 of rifampicin; ACAT, Advanced Compartmental Absorption & Transit model.

The study employs a comprehensive workflow to model and anticipate DDIs driven by CYP3A4 induction. The process begins with the development of a PBPK model for the inducing agent, rifampicin, which is meticulously validated against empirical human PK data. Following this, a PBPK model for the substrate drug is meticulously crafted, utilizing human PK and mass balance data to ensure model accuracy. In the subsequent phase, a PBPK-DDI model is meticulously formulated, as illustrated in Figure 1, to project the interaction profile between rifampicin and the substrate drug. The predictive accuracy of this model is rigorously evaluated by comparing its predictive accuracy with actual DDI data. Finally, to benchmark the performance, a traditional static model is applied to predict DDIs, and its predictions are critically compared with those from the PBPK model, thereby facilitating an assessment of the comparative efficacy and applicability of these methodologies.

### PBPK model of rifampicin and model validation

The construction of the rifampicin PBPK model was developed on the integration of core physico-chemical and biopharmaceutical attributes, including aqueous solubility, logarithm of the octanol-water partition coefficient (logD), fraction unbound in plasma (*f*_*up*_), and red blood cell partitioning (R_bp_). The human effective permeability (P_eff_) was meticulously calibrated to correspond with the absorption phase of the observed PK profile, with residual model parameters either prognosticated through the ADMET Predictor module or derived from the default settings within the GastroPlus simulation platform.

In the present investigation, PK parameters pertaining to the compartmental disposition were determined utilizing the PKPlus module of GastroPlus, calculated on the observed plasma concentration-time (C-T) profiles after intravenous administration. The system clearance included 1) the hepatic non-saturated metabolism mediated by CYP3A4 (about 0.0179 L/h/kg), which was defined in the Enzyme Table of GastroPlus using K_m_ and V_max_ of CYP3A4; 2) the remained liver metabolism (0.0623 L/h/kg), which was defined as the linear clearance in the Pharmacokinetics of GastroPlus; 3) and the renal clearance (0.0169 L/h/kg), which was defined as the clearance by glomerular filtration (GFR) based on the result of f_up_·GFR.

The induction potency (EC_50_) and the magnitude (E_max_) of rifampicin’s effect on CYP3A4 were extracted from the published data. The validation of the PBPK model was executed by juxtaposing the simulated plasma C-T profiles of rifampicin against empirical data following a 600 mg intravenous dose, a 400 mg single oral dose, and a 600 mg single and multiple oral doses. A comparative analysis of the PK parameters of rifampicin was also undertaken to substantiate the fidelity of the model.

### PBPK model of victim drugs and model validation

A total of 28 small molecule drugs approved by the FDA were selected as “victim” drugs for this study. These cases were culled from a previous investigation conducted by our research team, with the exclusion of those lacking reported interactions with rifampicin (Ren et al., 2021). The PBPK models for these victim drugs were constructed with detailed parameters sourced from the literature. Notably, these models incorporated the fraction metabolized (*f*_*m*_) parameter, which represents the proportion of a drug metabolized by CYP3A4. The determination and integration of the *f*_*m*_ parameter are detailed in the referenced literature (Jiang et al., 2023).

Each of these PBPK models was rigorously validated using human PK data and mass balance data. The validation process ensured the models’ fidelity to empirical observations. For further scrutiny, the validation datasets and detailed analyses are provided in the Supplementary Material.

### Software and data analysis

The PBPK modelling and simulations of victim drugs and perpetrator (rifampicin), as well as the DDI predictions, were conducted using GastroPlus™ Software (version 9.7; Simulations Plus, Inc., Lancaster, CA, United States). The C_max_ and AUC were calculated by the PKPlus module of GastroPlus.

### Static mechanistic models

In addition to the PBPK-DDI models, static mechanistic models were employed to predict DDIs, adhering to the guidelines outlined in “*In Vitro* Drug Interaction Studies: Cytochrome P450 Enzyme- and Transporter-Mediated Drug Interactions for Industry.” The steady-state DDI predictions, as default in GastroPlus, facilitate the generation of a comprehensive suite of DDI outcomes (Einolf et al., 2014). This includes a tabulation of the predicted plasma AUC ratios in the presence and absence of the perpetrator drug for each specified concentration. The models delineate the individual contributions of the gut and liver to the overall DDI, alongside the projected total change in AUC. The accuracy of these predictions is evaluated by comparing the anticipated AUCR with empirically derived ratios. Furthermore, the performance of the static mechanistic models is juxtaposed with that of the PBPK-DDI model, thereby highlighting the superior predictive capabilities of the PBPK-DDI model.

### Evaluation of predictive performance

The predictive accuracy of the model was assessed by comparing the predicted ratios of the AUC and C_max_ of the victim drug when co-administered with rifampicin to those observed when the victim drug was administered alone. Specifically, the AUCR and C_max_R were calculated. The predictive performance was deemed acceptable when the model’s projected ratios were within a two-fold deviation of the observed Geometric Mean Ratio (GMR), with the acceptable range defined as 0.5 to 2 times the GMR. Additionally, the methodology reported by Guest was employed to further evaluate the predictive efficacy of model (Guest et al., 2011).

## Results

### PBPK model development and validation of rifampicin

**FIGURE 2:**
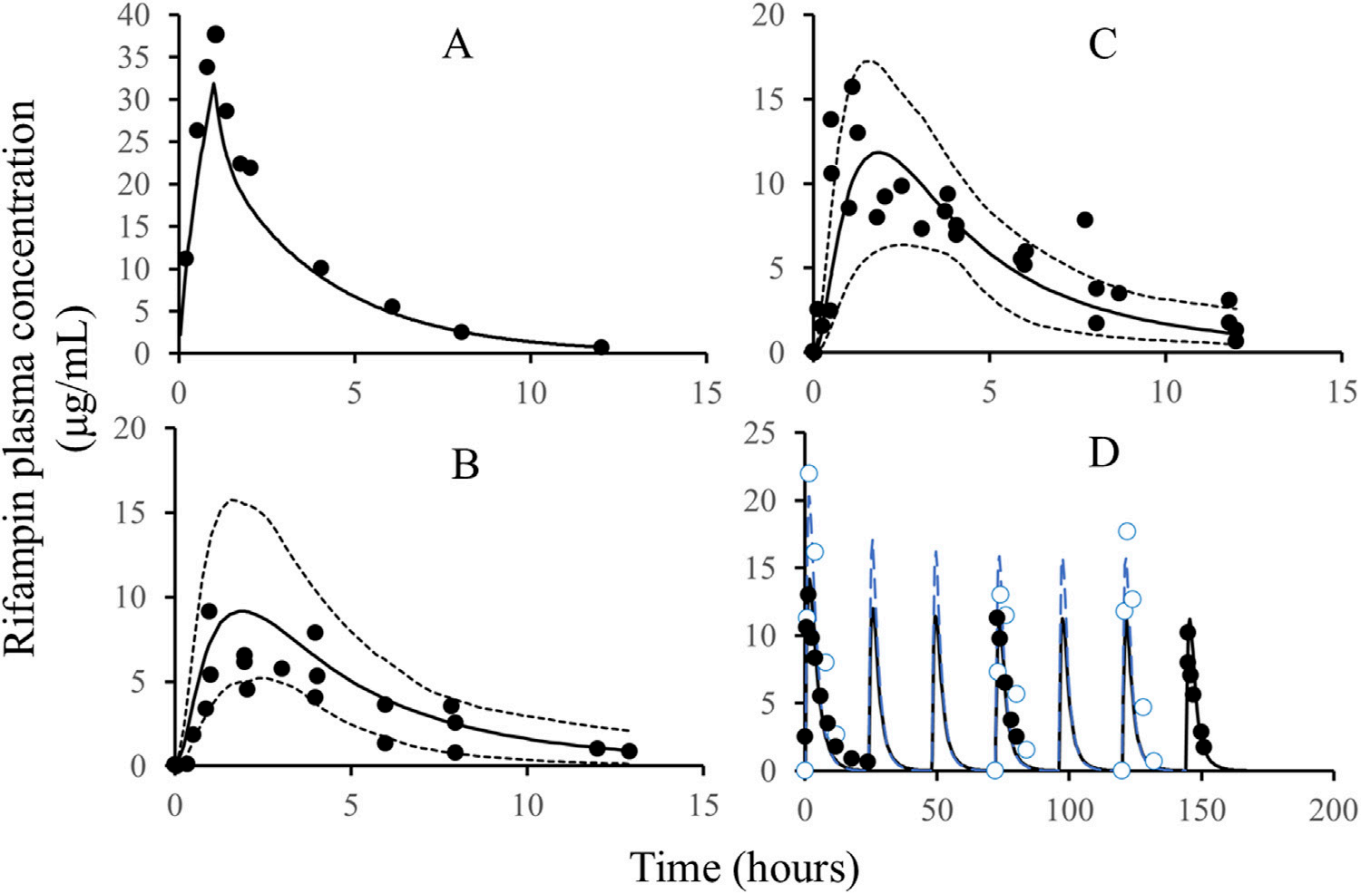
The comparison of simulated rifampicin PK in PBPK model with observed values. Note: Solid line: simulated C-T profiles of rifampicin after IV administration, single dose, 600 mg **(A)**, oral administration, single dose, 200 mg **(B)**, oral administration, single dose, 400 mg **(C)**, and oral administration, QD, 600 mg; dash line: quantile of 5% and 95%; black circles: the observed PK data of rifampicin; blue circles **(D)**: the observed PK data of rifampicin; blue line **(D)**: the simulated C-T profiles of rifampicin. The black and blue C-T profiles in Figure D are from two different publications with different demographic characteristics, such as body weight, height, and age.

The development of the rifampicin PBPK model was guided by a comprehensive set of physiological and physicochemical parameters, as detailed in Table 1. Utilizing these parameters, we simulated various dosing regimens to predict the human C-T profiles. The simulated C-T curves were compared with observed data, as depicted in Figure 2. The comparison revealed that the predicted curves closely matched the observed data points, indicating a high degree of fidelity in our model.

**TABLE 1:** Key parameters in rifampicin PBPK model.

Parameters	Value	Note
*Physicochemical Parameters*		
Molecular Weight (g/mol)	822.96	Baneyx et al. (2014)
LogD	1.3 (@ pH 7.4)	Baneyx et al. (2014)
pKa base (acid)	7.9 (1.7)	
Aqueous Solubility (mg/mL)	1.1 (@ pH 6.5)	
Biorelevant Solubility (mg/mL)	NONE	
Particle Radius (μm)	25	Defaulted value
Precipitation Time (s)	900	Defaulted value
P_eff_ (cm/s*10^4^)	1	Fitted data according to the observed PK
*Distribution Parameters*		
R_bp_	0.52	Baneyx et al. (2014)
*f*_*up*_ (%)	16	Baneyx et al. (2014)
V_c_ (L/Kg)	0.15145	Calculated values using PKPlus module based on the observed intravenous PK
K_12_ (1/h)	1.4219	
K_21_ (1/h)	1.7949	
*Elimination Parameters*		Hepatic metabolism mediated by CYP3A4
K_m_ (Gut & Liver, mg/L)	0.028	Baneyx et al. (2014)
V_max_ (Gut & Liver, mg/s)	13.33	Parameter defined in the record named “Rifampicin-PBPK” of GastDDIStandards.mdb
Other liver CL (L/h/kg)	0.0623	
Renal CL (L/h/kg)	0.0169	
*Induction Parameters*		
EC_50_ (μM)	0.8	Baneyx et al. (2014)
E_max_	14.6	Baneyx et al. (2014)

LogD, common logarithm of the octanol: water partition coefficient; pKa, -log10Ka, where Ka is acid dissociation constant; P_eff_, effective permeability; R_bp_, ratio of concentration in whole blood vs. plasma; f_up_, fraction unbound in plasma; V_c_, volume of distribution; K_12_, Rate constant for transfer from central to peripheral compartment; K_21_, Rate constant for transfer from peripheral to central compartment; K_m_, Michaelis-Menton constant; V_max_, maximum rate of drug metabolism or transport; CL, Clearance; EC_50_, Induction potency to CYP3A4; E_max_, Induction magnitude to CYP3A4.

To quantitatively assess the accuracy of our model, we calculated the parameters for both the observed and simulated curves using a non-compartmental analysis method. The results of this comparison are presented in Table 2. The predicted error of the parameters was found to be within the range of ±25%, which aligns well with the bioequivalence criteria (>0.8 and <1.25). This level of accuracy confirms the reliability of the rifampicin model and justifies its use for further predictions of DDIs involving CYP3A4 substrates.

**TABLE 2:** The comparison of predicted PK parameters with observed values for rifampicin.

PK parameters	Predicted	Observed	Predicted error/%
600 mg Rifampicin, single dose after intravenously administration			
C_max_ (μg/mL)	31.9	37.6	−15.16
T_max_ (h)	1	1.06	−5.66
AUC (μg·h/mL)	93.73	114	−17.78
V (L)	18.28	13.67	33.72
CL (L/h)	6.24	5.15	21.17
T_1/2_ (h)	2.22	2.21	0.45
200 mg Rifampicin, single dose after oral administration			
C_max_ (μg/mL)	8.92	10.3	15.59
T_max_ (h)	1.98	1.7	−14.57
AUC (μg·h/mL)	49.6	60	20.94
V/F (L)	44.24	50.88	−13.05
CL/F (L/h)	7.9	10.49	−24.69
T_1/2_ (h)	3.63	2.74	32.32
400 mg Rifampicin, single dose after oral administration			
C_max_ (μg/mL)	11.53	8.71	−24.5
T_max_ (h)	1.92	1.85	−3.77
AUC (μg·h/mL)	59.41	41.56	−30.05
V/F (L)	46.03	42.12	9.29
CL/F (L/h)	8.94	7.81	14.54
T_1/2_ (h)	3.37	2.95	14.24
600 mg Rifampicin, after oral administration, QD-1			
C_max_-Day 1 (μg/mL)	14.2	13	9.23
AUC-Day 1 (μg·h/mL)	70.34	85.63	−17.86
V/F-Day 1 (L)	35.27	61.44	−42.59
CL/F-Day 1 (L/h)	8.52	6.23	36.76
T_1/2_-Day 1 (h)	2.54	11.54	−77.99
C_max_-Day 4 (μg/mL)	11.3	11.3	0
AUC-Day 4 (μg·h/mL)	43.74	48.43	−9.68
V/F-Day 4 (L)	44.76	50.74	−11.79
CL/F-Day 4 (L/h)	12.74	9.86	29.21
T_1/2_-Day 4 (h)	2.06	3.43	−39.94
C_max_-Day 6 (μg/mL)	11.2	10.2	9.8
AUC-Day 6 (μg·h/mL)	41.24	34.05	21.12
V/F-Day 6 (L)	44.24	54.92	−19.45
CL/F-Day 6 (L/h)	12.96	15.98	−18.9
T_1/2_-Day 6 (h)	1.92	1.4	37.14
600 mg Rifampicin, after oral administration, QD-2			
C_max_-Day 1 (μg/mL)	20.3	22	−7.73
AUC-Day 1 (μg·h/mL)	89.34	132.27	−32.46
V/F-Day 1 (L)	23.9	21.98	56.53
CL/F-Day 1 (L/h)	6.59	4.21	−22.9
T_1/2_-Day 1 (h)	2.02	2.62	−4.11
C_max_-Day 4 (μg/mL)	15.9	13	22.31
AUC-Day 4 (μg·h/mL)	57.55	86.76	−33.67
V/F-Day 4 (L)	32.47	33.86	56.4
CL/F-Day 4 (L/h)	10.26	6.56	5.24
T_1/2_-Day 4 (h)	2.21	2.1	56.53
C_max_-Day 7 (μg/mL)	15.8	17.7	−10.73
AUC-Day7 (μg·h/mL)	57	96.73	−41.07
V/F-Day 7 (L)	32.48	25.26	28.58
CL/F-Day 7 (L/h)	10.36	6.11	69.56
T_1/2_-Day 7 (h)	2.2	1.46	50.68

Given the model’s validated predictive capacity, it will be employed in subsequent analyses to predict the interactions between rifampicin and various CYP3A4 substrates, providing a robust foundation for our predictions.

### PBPK models of victim drugs and their validation

The models for the victim drugs were meticulously constructed and validated using a combination of human data and mass balance data. These models were designed to simulate the disposition of drugs in the human body, accounting for absorption, distribution, metabolism, and excretion processes.

The accuracy of the victim drug models was assessed by comparing the simulated profiles and mass balance data with empirical observations. The results of this validation process demonstrated a high degree of consistency between the simulated and observed data, confirming the predictive power of our models.

For a detailed analysis and a comprehensive set of validation data, readers are directed to the Supplementary Material and our previously published articles (Ren et al., 2021), which lay a foundation for their application in predicting DDIs involving CYP3A4.

### Predictive performance of the PBPK-DDI model

**FIGURE 3:**
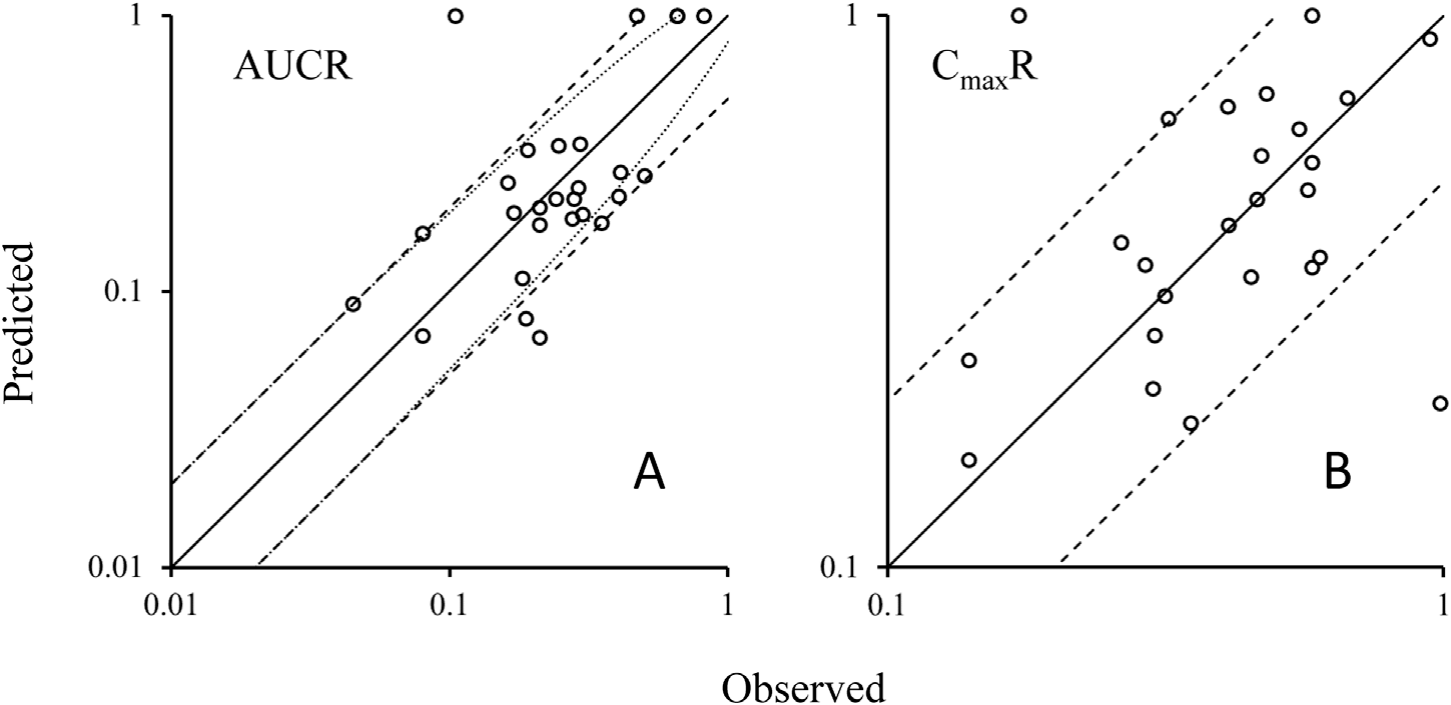
Plots of predicted AUCRs **(A)** and C_max_R **(B)** versus the observed values. Note: Solid lines represent the unit line (Y = X). The dash lines are the 0.5 fold and 2 fold lines; The dot lines are the lines using Guest-limit criteria. The circles represent the observed means of AUCR and C_max_R. AUCR, the ratio of area under the concentration-time curve of victim after co-administration with rifampicin over area under the C-T curve of victim with dosing alone; C_max_R, the ratio of victim C_max_ after co-administration with rifampicin over victim C_max_ with dosing alone.

Utilizing the validated model parameters for rifampicin and its substrates, coupled with the characterized E_max_ and EC_50_ values indicative of rifampicin’s induction potency on CYP3A4, we projected the potential DDIs. The predictive metrics centered on the exposure ratios, quantified by the AUCR and the C_max_R. The predictive performance of our PBPK-DDI model was rigorously assessed by comparing predicted AUCR and C_max_R with empirical data. This comparison is visually represented in Figure 3A, which delineates the alignment between predicted and observed AUC ratios. The analysis revealed a substantial concordance between predicted and observed ratios, with 89% of the predictions falling within the acceptable range according to the 0.5 to 2-fold criterion. When subjected to the Guest criteria, our model demonstrated a commendable alignment, with 79% of the predictions matching the observed data. Furthermore, to provide a more granular analysis, we specifically evaluated the concordance between the predicted and observed C_max_ ratios. As depicted in Figure 3B, an impressive 93% of the predicted C_max_R values agreed with the empirical C_max_R values within the 0.5 to 2-fold acceptance criterion. These findings underscore the robustness of our PBPK-DDI model in forecasting the clinical implications of CYP3A4-mediated DDIs induced by rifampin. The high predictive concordance, as evidenced by the adherence to both evaluation standards, affirms the model’s reliability and its potential utility in the realm of drug development and clinical pharmacology.

### Comparison of predictive performance with static model

The static model, a widely recognized and straightforward approach for evaluating DDIs as per DDI guidance, was employed for comparative analysis with our PBPK-DDI model. The comparison, detailed in Table 3, reveals significant discrepancies in the predictive accuracy of the Static model, particularly when assessing induction-based DDI risks.

**TABLE 3:** The Dosing method of victim drugs and the predictive performance using different approaches.

Victims	Dosing method	AUCR			Predicted/Observed		References
		Observed	Predicted				
			Static model	PBPK	Static model	PBPK	
Abiraterone	1,000 mg, SD	0.42	0.04	0.22	0.08	0.51	Bernard et al. (2015)
Apixaban	10 mg, SD	0.47	1.00	1.00	2.13	2.13	Vakkalagadda et al. (2016)
Apremilast	30 mg, SD	0.28	0.04	0.22	0.14	0.78	Liu et al. (2014)
Axitinib	5 mg, SD	0.21	0.02	0.17	0.10	0.83	Pithavala et al. (2010)
Baricitinib	10 mg, SD	0.66	1.00	1.00	1.53	1.53	(FDA, 2018)
Bosutinib	500 mg, SD	0.06	0.01	0.07	0.23	1.15	Abbas et al. (2015), Ono et al. (2017)
Crizotinib	250 mg, SD	0.18	0.02	0.11	0.12	0.62	Xu et al. (2015), Yamazaki et al. (2015)
Edoxaban	60 mg, SD	0.66	1.00	1.00	1.52	1.52	Mendell et al. (2015)
Flibanserin	100 mg, SD	0.05	0.01	0.09	0.28	1.80	(FDA, 2015)
Fostamatinib	150 mg, SD	0.25	0.10	0.34	0.40	1.35	Martin et al. (2016)
Ibrutinib	560 mg, SD	0.10	0.05	0.07	0.49	0.72	de Jong et al. (2015); de Zwart et al. (2016)
Lenvatinib	24 mg, SD	0.91	1.00	1.00	1.10	1.10	Shumaker et al. (2014)
Macitentan	^a^10 mg, QD	0.21	0.01	0.07	0.05	0.32	Bruderer et al. (2012), de Kanter et al. (2016)
Nintedanib	150 mg, SD	0.50	0.10	0.26	0.20	0.52	Luedtke et al. (2018)
Ospemifene	60 mg, SD	0.41	0.05	0.27	0.12	0.66	Lehtinen et al. (2013)
Panobinostat	20 mg, SD	0.35	0.01	0.18	0.03	0.51	Einolf et al. (2017a)
Ponatinib	45 mg, SD	0.41	0.02	0.22	0.05	0.54	Morita and Hanada (2022), Narasimhan et al. (2015)
Roflumilast	500 ug, SD	0.19	0.11	0.33	0.58	1.72	Jia et al. (2024), Nassr et al. (2009)
Rolapitant	180 mg, SD	0.17	0.01	0.19	0.06	1.14	(FDA, 2017)
Ruxolitinib	50 mg, SD	0.29	0.05	0.34	0.17	1.18	Shi et al. (2012), Shi et al. (2015), Umehara et al. (2019)
Sonidegib	800 mg, SD	0.21	0.02	0.18	0.10	0.88	Einolf et al. (2017b)
Tasimelteon	20 mg, SD	0.11	0.02	0.15	0.17	1.35	Ogilvie et al. (2015)
Telaprevir	750 mg, SD	0.08	0.01	0.16	0.15	2.03	Garg et al. (2013)
Tofacitinib	30 mg, SD	0.16	0.02	0.25	0.13	1.55	Nam et al. (2020)
Venetoclax	200 mg, SD	0.29	0.02	0.24	0.05	0.82	Agarwal et al. (2016), Freise et al. (2017)
Vorapaxar	^a^20 mg, QD	1.08	0.11	0.80	0.10	0.74	Kosoglou et al. (2013)
Fedratinib	500 mg, SD	0.19	0.02	0.08	0.08	0.42	Ogasawara et al. (2021)
Istradefylline	40 mg, SD	0.21	0.01	0.20	0.06	0.96	Mukai et al. (2018)
Percentage within 0.5∼2 fold					14%	89%	
Percentage within Guest criteria					11%	79%	

^a^ The DDIs, were evaluated by the PK, of victims at steady state with or without rifampicin.

Upon evaluation, it was observed that the Static model significantly overestimated the DDI risk in 83% of the cases, with only 14% of the predicted AUCR falling within the empirically accepted range of 0.5–2 times the observed AUCR. When applying the more stringent Guest criteria, this predictive accuracy further declined to 11%.

This comparative analysis underscores the Static model’s limitations in accurately forecasting DDIs, particularly when compared to the superior predictive capabilities of the PBPK-DDI model reported in this study. The PBPK-DDI model’s enhanced predictive performance is attributed to its physiologically based, mechanistic approach, which more accurately captures the complex interplay of drug interactions in the human body.

## Discussion

This study presents a comprehensive evaluation of the PBPK model’s predictive capabilities for DDIs mediated by CYP3A4 induction. Through meticulous model development and validation, we have demonstrated the model’s high fidelity in simulating the complex profiles of both rifampicin and its substrates. The comparison with empirical data has confirmed the model’s robustness, with an impressive alignment between predicted and observed parameters, well within the accepted bioequivalence criteria. Furthermore, the predictive performance of our PBPK-DDI model significantly surpassed that of the conventional Static model, particularly in accurately estimating the risks associated with CYP3A4 induction. This superiority is evident in the model’s ability to closely mirror the clinical outcomes, as evidenced by the high percentage of accurate predictions based on both the 0.5 to 2-fold and Guest criteria. The findings underscore the transformative potential of the PBPK-DDI model in enhancing the safety and efficacy assessments of new molecular entities during early clinical development.

Our rifampicin PBPK model, informed by the comprehensive framework within GastroPlus’s full PBPK model, has been strategically simplified to a compartmental model for enhanced practicality and expedited application. This tailored approach, while retaining the physiological essence of the full model, allows for rapid predictions that are crucial in clinical and research settings. Our model parameters, fine-tuned against a spectrum of published literature and aligned with empirical human data, have been optimized to ensure the most accurate predictions, as evidenced by their close match with observed plasma C-T profiles (Acocella, 1978; Loos et al., 1985; Peloquin et al., 1997). This validation not only affirms the model’s reliability but also positions it comparably with other published rifampicin PBPK models, reinforcing the consensus on model parameters that best capture the drug’s behavior (Ono et al., 2017; Yamazaki et al., 2015; de Zwart et al., 2016; Luedtke et al., 2018; Morita and Hanada, 2022; Nassr et al., 2009; Umehara et al., 2019; Freise et al., 2017).

The robustness for the PBPK model of victim drugs is crucial for assessing the DDIs. Our models align with existing scientific literature and highlight the significance of the *f*_*m*_ parameter. By incorporating empirically derived *in vitro f*_*m*_ values into our models, we prevent the potential overestimation of DDI risk that could occur if we assumed a *f*_*m*_ value of 100%. Although this nuanced approach lacks comparative data in this presentation, it is theoretically sound and enhances the precision of DDI predictions, aligning with the current scientific consensus on this topic.

In our study, we observed a significant discrepancy in the *f*_*m*_ test results for tasimelteon when using microsomal and recombinant enzyme methods. The microsomal method, after multiple repetitions, yielded results ranging from 0% to 15%, while the recombinant enzyme method resulted in a 65% value. The cause of this difference may be attributed to non-enzymatic metabolism in the microsomes; further investigation is underway to pinpoint the exact reason. According to the submission information on tasimelteon published by the FDA (FDA, 2014), CYP3A4 is the primary enzyme involved in the metabolism of tasimelteon. Therefore, in this research, we have adopted the results based on the recombinant enzyme method.

Our current model demonstrates a high degree of predictive accuracy for DDIs, the multifaceted nature of *in vivo* DDIs suggests that there is still room for refinement. One of the factors that may influence the prediction of rifampicin’s inducing effect is its ability to induce multiple enzymes while also inhibiting transporters, potentially leading to increased drug absorption. This phenomenon is particularly evident with elagolix (FDA, 2020), where co-administration with rifampicin does not reduce but rather increases its exposure, highlighting the significant contribution of transporters over the inhibition of metabolic enzymes. Our model does not currently integrate the impact of transporters, which is why we have not included elagolix as a case study. The examples we have utilized are mostly drugs with minimal transporter involvement, which to some extent, has bolstered the precision of our predictions. Future models could benefit from incorporating the effects of transporters to further enhance the accuracy of DDI predictions.

## Conclusion

The PBPK models have effectively predicted DDIs involving rifampicin, highlighting their utility in clinical drug development. The models’ alignment with empirical data confirms their reliability, and their refinement will further enhance predictive precision in future studies.

## Data Availability

The original contributions presented in the study are included in the article/Supplementary Material, further inquiries can be directed to the corresponding authors.

## Abbreviations

AUCR, the ratio of area under the concentration-time curve of victim after co-administration with rifampicin over area under the concentration-time curve of victim with dosing alone; C_max_R, the ratio of victim C_max_ after co-administration with rifampicin over victim C_max_ with dosing alone; FDA, Food and Drug Administration; *f*_*m*_, the fraction of metabolism; Fa, cumulative intestinal absorption, %; V_c_, distribution volume in central compartment; K_12_, transfer rate constant from central compartment to peripheral compartment; PC1, peripheral compartment 1; PC2, peripheral compartment 2.
