# Supplementary material for: High-performance PBPK model for predicting CYP3A4 induction-mediated drug interactions: a refined and validated approach
Source: Front Pharmacol. 2025 Feb 26;16:1521068. doi: 10.3389/fphar.2025.1521068 (PMC11897275; doi:10.3389/fphar.2025.1521068)
Supplement: Supplementary file 1 [file Supplementaryfile1.docx]

**Validation of the victims PBPK Model**

Absorption

To simulate oral drug absorption, we primarily utilized the ACAT (Advanced Compartmental Absorption & Transit) model. The ACAT model is a comprehensive compartmental modeling approach that considers the complex dynamics of drug absorption and transit through the gastrointestinal (GI) tract. This model integrates the dissolution model, permeability calculations, and physiological parameters within the GastroPlus software to simulate the intricate processes involved in oral drug absorption. The dissolution model was employed to simulate the drug's dissolution in GI fluids. This model takes into account factors such as drug solubility and pH, providing insight into the dissolution rate and availability of the drug for absorption. The permeability of the drug following dissolution in the GI tract considers various permeability mechanisms, including transcellular transport and paracellular diffusion. In vitro apparent permeability data from Caco-2 cell experiments were combined with the permeability model to estimate the drug's permeability across the GI tract. Essential physiological parameters were factored into the ACAT model to better predict oral drug absorption. These parameters include: ⅰ) Gastrointestinal transit time. ⅱ) Surface area: The available surface area within the GI tract for drug absorption. ⅲ) Blood flow rate.

The primary factors affecting absorption are solubility and permeability. To assess solubility and permeability, we gathered both qualitative (such as low, median, and high solubility or permeability） and quantitative data (solubility at different pH value and P_appA-B_ value in Caco-2). Considering the discrepancies between *in vitro* and *in vivo* environments, we didn’t use the quantitative *in vitro* data directed, but restricted the parameter settings related to solubility and permeability in the PBPK model to a qualitative perspective only. For the validation of the absorption model, we employed *in vivo* Fa (fraction absorbed): Firstly, we obtained data from mass balance studies using radiolabeled substances, specifically determining the proportion of drug excreted in feces. Using this data, we evaluated the Fa value, defined as Fa = 1 minus the proportion of unchanged drug excreted in feces. Finally, we compared the simulated Fa values with the measured Fa values, as illustrated in the Figure below.


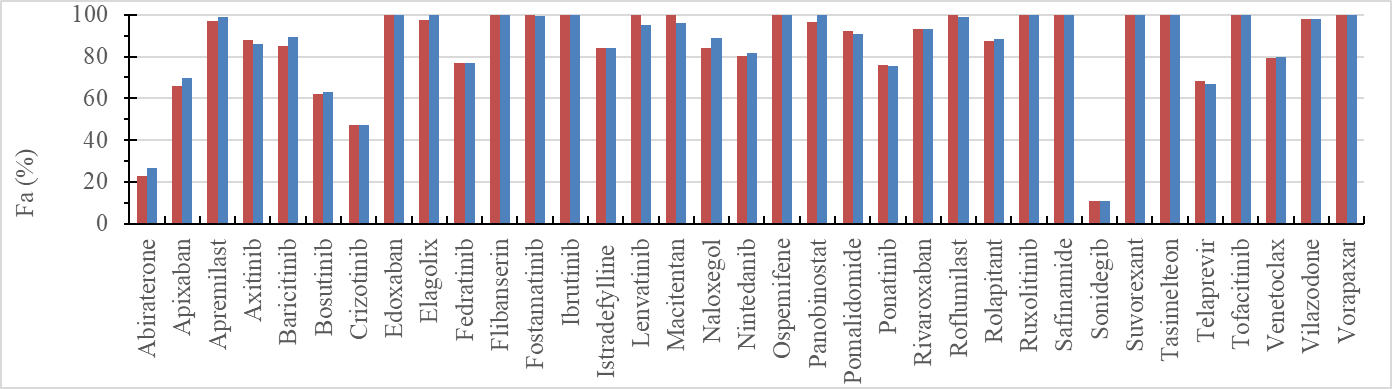


Figure S1. The comparison of Fa values between the simulated (blue column) and observed (red column).

Elimination (Metabolism and excretion)

Firstly, since fecal excretion has already been validated through Fa determination for the unchanged drug, we will not elaborate on it further at this point.

Secondly, for renal excretion, we can obtain the cumulative excretion proportion of the unchanged drug through the kidneys and use it to compare and validate the simulated renal excretion in the model. Please find the comparison of simulated renal excretion and actual renal excretion in the following table:


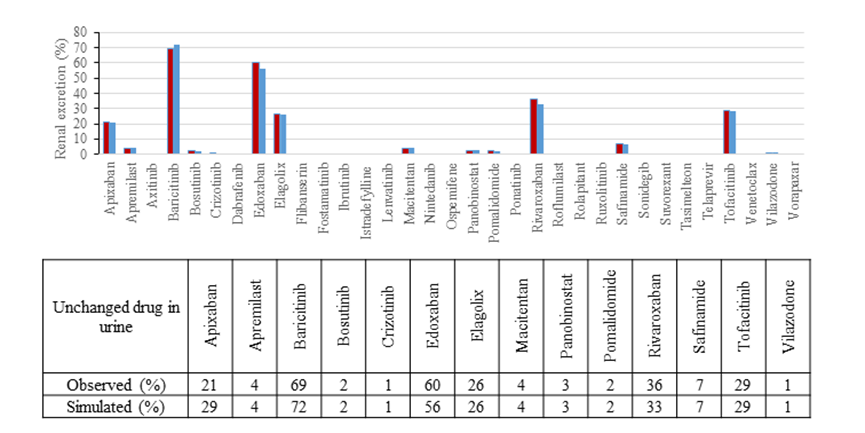

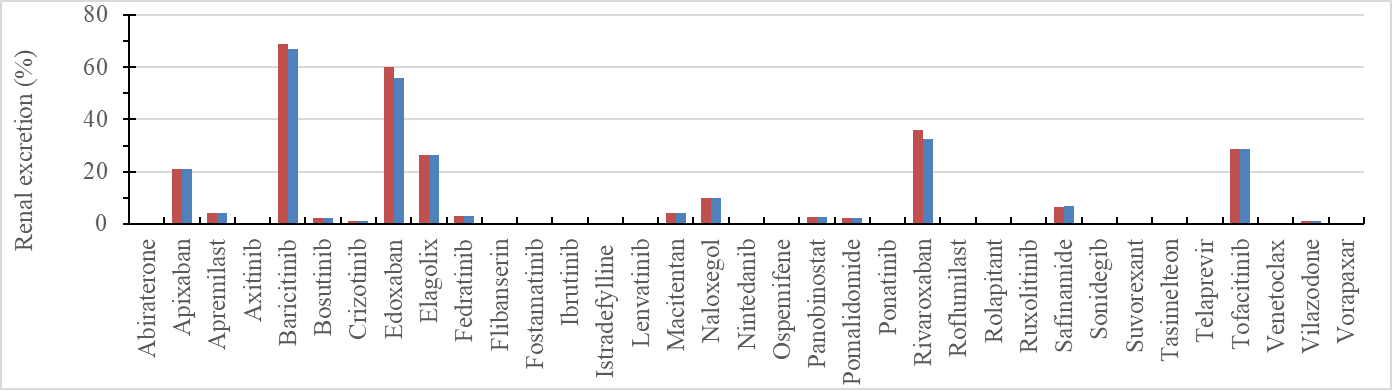


Figure S2. The comparison of renal excretion values between the simulated (blue column) and observed (red column). The table presented below the figure summarizes the renal excretion data for drugs exhibiting renal elimination characteristics. For drugs not featured in the table, both observed and simulated renal excretion values for the parent compound are reported as 0.

Finally, we assume the absence of extrahepatic metabolism, with all metabolism occurring within the liver and intestines. The distribution of the drug in tissues is reversible, ultimately returning to the plasma. Thus, assuming fixed excretion parameters, there should be a strong relationship between the metabolism of the drug in the liver and the level of the unchanged drug present in the plasma. Please find the comparison of the simulated AUC with actual AUC in the following Figure:


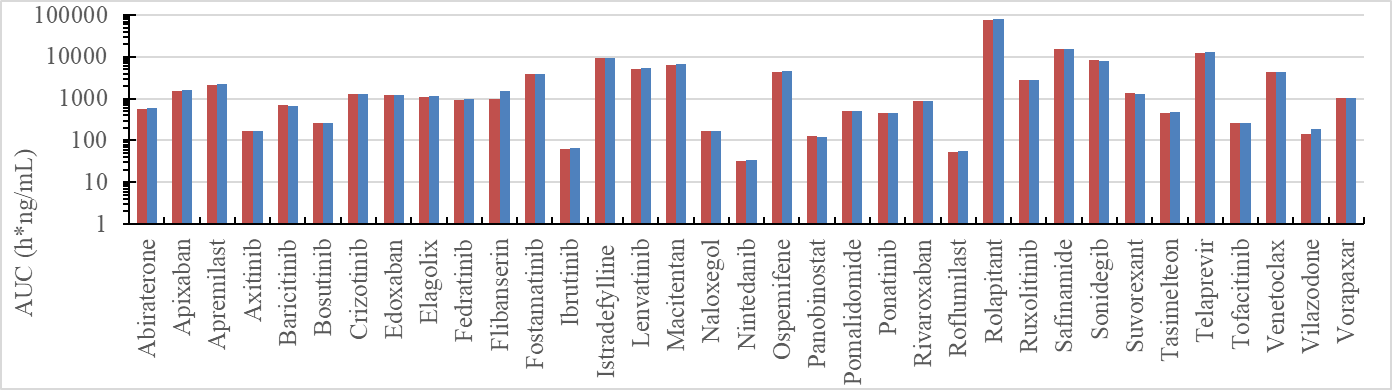


Figure S3. The comparison of renal excretion values between the simulated (blue column) and observed (red column).

Distribution:

In our predictions, we utilized a compartmental modeling approach, specifically the two-compartment model. This model includes distinct compartments representing key physiological sites such as the intestine, liver, central compartment, and peripheral compartment. The two-compartment model provides a simplified yet comprehensive representation of drug distribution dynamics within the body.

Given the utilization of the two-compartment model, the concepts of perfusion-limited and permeability-limited distribution mechanisms are not directly applicable. Instead, the model inherently accounts for the complexities of drug distribution by characterizing the movement of drugs between the central and peripheral compartments, which represent highly perfused and less perfused tissues, respectively.

In this model, we have implemented two approaches to ensure a distribution profile that closely resembles real-world conditions.

Firstly, we have incorporated measured values for distribution-related parameters such as plasma protein binding and blood-to-plasma ratio (BP ratio). This enables us to align these parameters in the model with empirically observed values.

Secondly, we consider distribution to be a reversible process. Therefore, the primary factor determining distribution is the drug's ability to return to the plasma after entering other compartments assuming fixed absorption, metabolism, and excretion. Consequently, the shape and distribution of the drug within the plasma exhibit greater relevance. The simulated PK profiles of victims are compared with the actual PK profiles in the following figure:


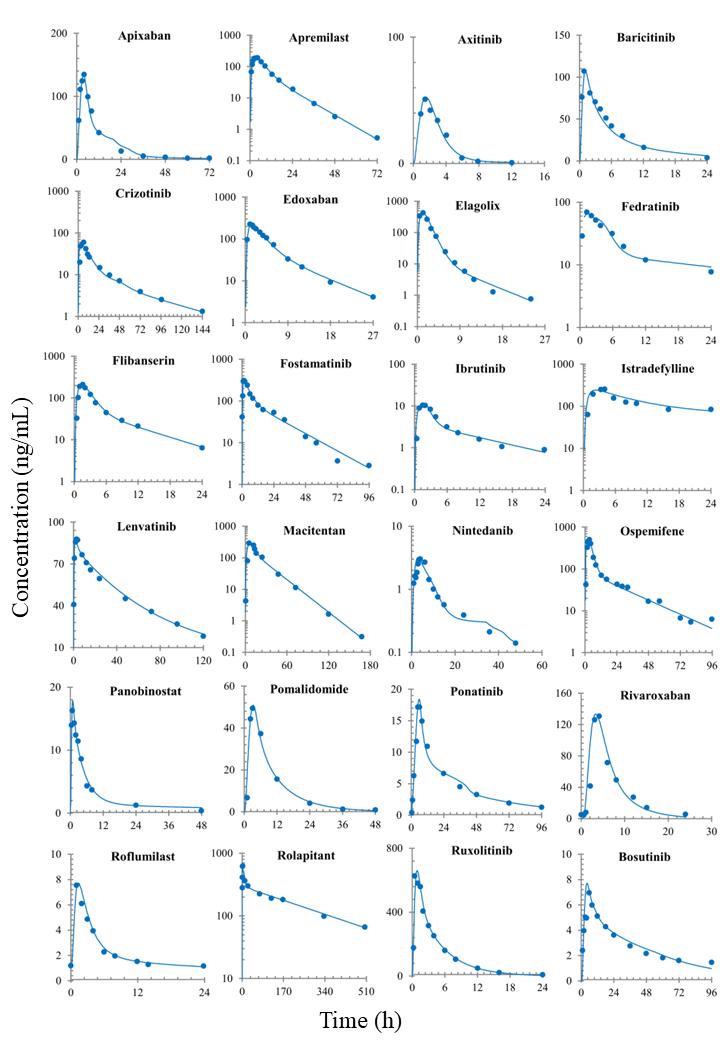


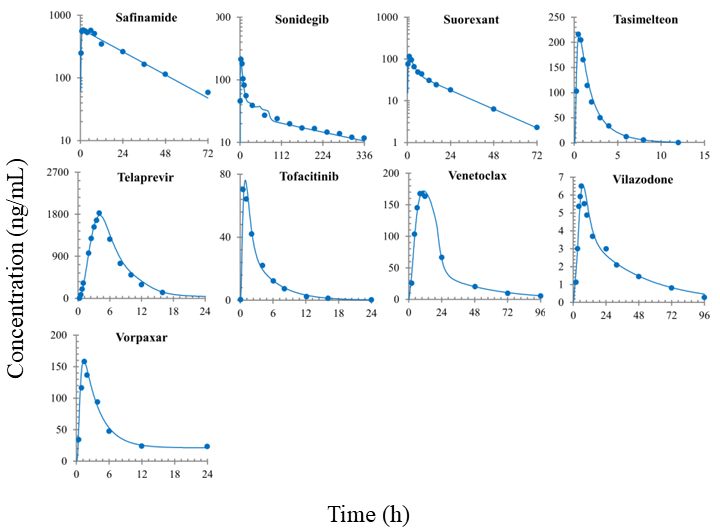


Figure S3. Model-simulated pharmacokinetic plasma profiles, following p.o. alone, overlaid with the observed data. Blue solid lines and circles represent the fitted pharmacokinetic profiles and the observed mean data after p.o. alone, respectively.

By above validations, we strived to achieve a more accurate representation of drug absorption, distribution, metabolism, and excretion within the model, closely resembling its behavior in real-world scenarios.
